# The Paintings

**Published:** 1872-06

**Authors:** 


### THE PAINTINGS

Were all sold yesterday. It was a magnificent collection. The rooms where they were displayed were filled before ten o'clock in the morning with men and women of cultivation and refinement, some coming to purchase, others to admire. Two months ago they were hanging in the splendid rooms of a country mansion, on which hundreds of thousands of dollars had been expended by the rich banker owner; but he was in prison now, and those elegant paintings, with vases of curious shape, and statuary of purest marble, by artists of the highest fame, collected with the greatest care and taste in the course of long years in travels at home and abroad, — these all were under the auctioneer's hammer, to be scattered over the land to increase other collections, destined sooner or later to similar dispositions; for all of earth is transitory, sparkling for a moment, then to die away forever in the darkness of the night of death.

But two months ago that owner sat in his counting-room in the very prime of full manhood and robust health, with his massive, well-built frame, looking "like a pine-knot," making thousands in a day, and sometimes in an hour. He was the soul of honor and of business integrity then; artistic, educated, cultivated, and refined. Few stood as high in social life or on 'change as he; and yet, for one little act, inconsiderate, it is true, still, most unfortunately, it was committed, and he was hurried off to prison, the

PRISON OF THE GRAVE!

He left his home in Fifth Avenue one evening to go to the opera; he might have called his carriage, but it was just after dinner, and he thought a little walk would do him good. Before he reached the place of music and of song, a sudden shower fell, and wetted his clothing. It did not seem much wet to him; he walked fast, and thought it would dry in the warm building, but it did not; instead, he felt a shiver pass over him. He did not like to lose the music, and concluded to sit it out, which he did. On returning home he felt uncomfortable; warmed himself, and went to bed, waking up in the early morning with

PNEUMONIA, and in two days he was dead; in two more, buried. There was a large funeral; the papers were full of his praises; they said no other man would be so much missed; that his place could scarcely be filled with one so frank and liberal and generous as he.

There were almost a hundred paintings, may be more. Three of them cost fifty thousand dollars; but money, and position, and culture, and high and noble qualities can purchase no exemption from the penalties of the violated laws of our being. Any man with a single mite of common sense ought to have known better than to sit still in damp clothing for two hours at a stretch; but he didn't, and died as the result.

